# Viewing images of snakes accelerates making judgements of their colour in humans: red snake effect as an instance of ‘emotional Stroop facilitation’

**DOI:** 10.1098/rsos.140066

**Published:** 2014-11-05

**Authors:** Masahiro Shibasaki, Tomoko Isomura, Nobuo Masataka

**Affiliations:** Primate Research Institute, Kyoto University, Kanrin, Inuyama, Aichi, Japan

**Keywords:** snake fear, colour perception, emotional Stroop interference, pictorial Stroop paradigm, enhancement of perception

## Abstract

One of the most prevalent current psychobiological notions about human behaviour and emotion suggests that prioritization of threatening stimuli processing induces deleterious effects on task performance. In order to confirm its relevancy, 108 adults and 25 children were required to name the colour of images of snakes and flowers, using the pictorial emotional Stroop paradigm. When reaction time to answer the colour of each stimulus was measured, its value was found to decrease when snake images were presented when compared with when flower images were presented. Thus, contrary to the expectation from previous emotional Stroop paradigm research, emotions evoked by viewing images of snakes as a biologically relevant threatening stimulus were found to be likely to exert a facilitating rather than interfering effect on making judgements of their colour.

## 2. Introduction

There have been increasing reports about influences exerted by emotion upon behaviour. In particular, the manner by which processing emotional visual stimuli produces these effects is of great interest. Concerning these issues, one of the notions prevalent among psychologists is that due to limited attentional capacity, prioritization of emotional stimuli processing induces deleterious effects on task performance [1,2]. Perhaps the most typical experimental testing of this notion has been performed using the emotional Stroop paradigm developed by psychopathologists [3–5]. In this paradigm, phobic participants are asked to make judgements of the colour of presented textual words that vary in personal, emotional significance. Delays in judgements of colour, or Stroop interference, has been said to occur when the naming of the word captures the participant's attention despite the participant's attempt to attend to its colour [6]. As a consequence, participants are likely to exhibit delayed colour-naming for words related to their current concerns, and these concerns are most often threat-related. Moreover, more anxious participants are likely to be slower at that colour-naming. This is considered to be related to the fact that reacting defensively in fearful situations is crucial to the survival of all animal species, including humans [7].

Indeed, most non-human primates are known to have evolved an innate predisposition to quickly associate fear with some specific threatening stimuli. This is typically the case for their response to venomous snakes [8]. In humans, too, snake-phobia is regarded as a phenomenon that is widespread throughout the world [9]. An even stronger version of such an argument was published recently [10]. In a comprehensive analysis of the origin of the human visual system, the author discussed the possibility that some of its basic properties evolved precisely because they facilitated the detection of snakes. Evidence supporting that notion included a series of investigations that showed that human adults have an attentional bias for the detection of fear-relevant stimuli such as snakes compared to neutral stimuli, such as flowers and mushrooms [8]. More recent studies have documented that preschool children, 8- to 14-month-old infants, and even non-human primates also detect snakes more quickly than flowers [11–15]. The existence of neurons that respond selectively to snake images has been reported in macaque monkeys [16].

Considering these findings, one may be led to reason from a psychopathological perspective that humans would need to spend more time to make judgements about the colour of images of snakes than they would need to make judgements about the colour of images of neutral stimuli such as flowers. However, given the recent findings that snake recognition can even occur in humans prior to awareness [9,11,13–15], the possibility of the opposite prediction arises; evolved dangerous stimuli should be more rapidly and less cognitively responded to. According to this hypothesis, one can predict that viewing images of snakes would accelerate making judgements of their colour. Darwin [17] has already claimed the possibility, stating that ‘fear often acts at first as a powerful stimulant’, and that ‘a man or animal driven through terror of desperation, is endowed with wonderful strength’. Indeed, suggestive empirical evidence for this hypothesis has been presented by the findings of a previous study concerning snake fear that were obtained with the textual emotional Stroop paradigm [18]. In the study, the participants with such biologically adaptive fear were tested on words related to snakes (e.g. *cobra*), to a general threat (e.g. *fail*), or to a neutral household theme (e.g. *spoon*). They were tested in the presence of a boa constrictor (and were told to see how close they could get to the snake). Under these conditions, the study did not find, within the colour-naming latency data, the predicted emotional Stroop interference in patients with anxiety disorders. These findings were confirmed by others later [19]. Based on these results, the present study was designed to determine which of the two predictions fit the data we would collect, using the pictorial emotional Stroop paradigm [20–22].

**Figure 1.**
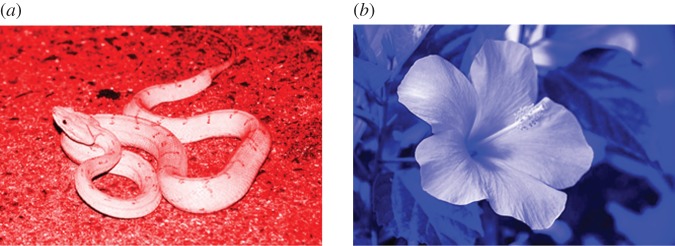
Examples of the stimuli used in the experiment. (*a*) Image of snake in red and (*b*) image of flower in blue.

In the experiment, reaction time (RT) was measured when participants named the colour of either an image of snakes or one of flowers, both of which varied in colour (either red, green or blue; figure 1). We decided to use this paradigm, because pictorial stimuli would be more effective or more ecologically valid than text for assessing influences exerted by viewing biologically relevant threatening stimuli. As reported below, the results indicate the fact that emotion (possibly fear) evoked by the images of snakes exerts a facilitating effect upon making judgements of their colour.

## 3. Material and methods

In this study, 108 undergraduates (mean age (s.d.) = 20.71 years (1.02)) from several universities in Aichi prefecture, Japan, were tested as the adult participants, and 25 schoolchildren between 7 and 13 years of age from several elementary schools located in the same region were tested as the participant group of children (mean age (s.d.) = 10.12 years (1.56)). They were all right-handed and naive as to the purpose of this study and had normal or corrected-to-normal vision.

As the stimuli, 20 photographs of snakes and 20 photographs of flowers were prepared. They were the same as those used in our previous snake detection studies [13–15]. Each of the 40 pictures appeared three times—once in red, once in green and once in blue—for a total of 120 experimental trials. These three colours were chosen because they have been extensively used as stimuli in previous psychological research regarding colour perception [23–25]. Moreover, they have been used extensively as standard stimuli in previous studies concerning Stroop effects [3,6,26,27]. To create colour-filtered images, we first removed all colour content from the photographs, generating black and white images. Subsequent colour balance manipulation along three different dimensions transformed the shadows, midtones and highlights of each greyscale image, thereby preserving luminosity (figure 1). Each stimulus was presented against a black background.

The experiment was conducted in several sound-attenuated rooms that were familiar to all of the participants, because they had already been tested there during our previous studies [15,25]. A 22-inch monitor connected with a personal computer was placed on a table. An adapted single-trial version of the pictorial emotional Stroop task [20] was used in the present experiment. Participants were told by an experimenter, who had not been notified about the purpose of this study, that they would see a series of colour-filtered pictures and should indicate the colour of each image as quickly as possible via key-press, while ignoring the content of each picture. During the experiment, they were required to put each of the index, middle and third finger of the right hand each onto one of three different keys on the external numeric keypad, and were instructed to press the index finger to indicate that the picture was red, to press the middle finger to indicate that the picture was green and to press the third finger for a blue image. The relationship between keypad and colour was counterbalanced across the participants.

Both practice and experimental trials consisted of three events: (i) a white fixation cross, which appeared at the centre of the screen for 1 s, (ii) the stimulus (picture), and (iii) a blank black screen (duration 0.8 s). Each stimulus remained on the screen until the participant pressed one of the three keys, and its duration was recorded as RT in each trial. The 15 practice and 120 experimental trials were randomized in advance. For the practice trials, one random sequence was generated.

We only analysed data from images matched to the correct colours. Mean error rates were 4.24% for the adult participants and 2.78% for the child participants.

## 4. Results

**Figure 2.**
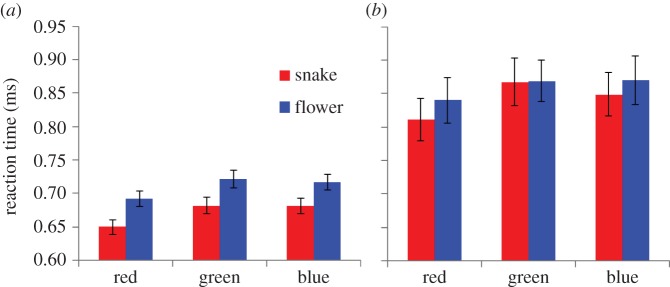
RTs of the participants. (*a*) Adult and (*b*) child participants across the six variations of the stimuli.

Figure 2*a*,*b* presents mean RTs of the adult and child participants to the six different stimuli. When the collected data were analysed by a 2 (adult/child, participant) × 2 (snake/flower, image) × 3 (red/green/blue, colour) ANOVA (analysis of variance), all of the three main effects were statistically significant (*F*_1,131_=35.74, *p*<0.001, *η*^2^_p_=0.214 for participant, *F*_1,131_=52.90, *p*<0.001, *η*^2^_p_=0.289 for image, *F*_2,262_=12.23, *p*<0.001, *η*^2^_p_=0.086 for colour). The interaction between participant and image was also significant (*F*_1,131_=8.05, *p*<0.005, *η*^2^_p_=0.059). However, the interaction between participant and colour was not significant (*F*_2,262_=0.26, *p*=0.772, *η*^2^_p_=0.002). The interaction between image and colour was not significant (*F*_2,262_=1.35, *p*=0.260, *η*^2^_p_=0.009) either. The interaction among participant, image and colour was not significant (*F*_2,262_=1.13, *p*=0.323, *η*^2^_p_=0.009). Subsequent analyses of simple main effects (Bonferroni correction), which were performed because of the significant interactions between participant and image, revealed that the RT to snake images was shorter than the RT to flower images both in adults (*p*<0.001) and in children (*p*<0.05) although the difference was more robust in the adults than in the children. The RT to red images was shorter than that to green images (*p*<0.001) or to blue images (*p*<0.001), whereas the RT to green images was not significantly different from the RT to blue images (*p*>0.10).

## 5. Discussion

Results of this study apparently present evidence against the prediction that viewing images of snakes interferes with making judgements of their colour. The data are consistent with the findings of the previous study [18,19] obtained using the textual Stroop paradigm and were found to fit the opposite prediction. The RT required to name the colour of an image of snakes was decreased when compared with when naming the colour of an image of flowers. In addition, the RT to name the colour of red images was shorter when compared with that for naming the colour of green or blue images. These findings about the relationship between RT and the colour of the stimuli were predictable because accumulating evidence has indicated that a stimulus presented in red draws attention more readily than the same stimulus presented in other colours, such as green or blue [28]. Nevertheless, in this study, viewing snake images, which can capture attention more readily than flower images, has facilitated naming the colour of those images. As a result, the participants had the most rapid responses to images of red snakes.

Apparently, the observed phenomenon could not be explained by ‘emotional Stroop interference’ [3], but rather can be referred to as ‘emotional Stroop facilitation’, and is consistent with the recent argument that emotion facilitates perception [29]. While that argument was based upon the results of a study in which a prior exposure to an image of a fearful face enhanced subsequent visual processing, the present findings indicate that this is the case for concurrent processing of the target as a threatening stimulus and making a judgement of its colour.

To the best of our knowledge, this study is the first attempt to systematically test a pictorial emotional Stroop paradigm, using snake images, except for one preliminary study [20]. The purpose of the preliminary study was to develop the experimental protocol. Since the number of the participants included in the study was limited, it is difficult to compare the present findings to that study. Nevertheless, they appear to be in accordance with the results of a previous study [6] that reported the acceleration of colour-naming in healthy students on exposure to an anxiety-producing film. Moreover, subsidiary data collected from healthy participants involved in a psychopathological study as a control group present evidence that strongly supports our findings [26]. The study showed that latency to colour-naming was longer for threatening words than for non-threatening words in patients with anxiety disorders, but that the latency was longer for non-threatening words than for threatening words in healthy adults. The authors of the study did not provide any explanation for the results. Taken together with these findings, however, the results of this study indicate that as far as healthy adults and children are concerned, they are more adept at avoidance when being exposed to evolved dangerous stimuli, by responding less cognitively. This is also in line with the notion stated by Darwin that emotions are adaptive insofar as they prompt actions beneficial to the organism [30]. Darwin [17] also noted fear such as that ‘…soon induces utter, helpless prostration, as if in consequence of, or in association with, the most violent and prolonged attempts to escape from the danger’. The biological structures that support these responses also usually communicate with those that provide the ability to inhibit and control reactions involving the cognitive processing of numerous attributes of a target as a threatening stimulus.

Finally, it should be noted that the emotional Stroop facilitation observed here was more robust in the adult participants than in the child participants. It is well known that children are often less fearful of snakes than adults [9,10]. Although that could partly explain the overall longer RTs in the children, the possible roles of factors such as experience, maturation and socialization that may have been operative are yet unknown. Further investigation into this issue is required in the near future.

## Data accessibility

Raw data: Dryad doi:10.5016/dryad.j22k0.
